# Socially Driven Consistent Behavioural Differences during Development in Common Ravens and Carrion Crows

**DOI:** 10.1371/journal.pone.0148822

**Published:** 2016-02-05

**Authors:** Rachael Miller, Kate L. Laskowski, Martina Schiestl, Thomas Bugnyar, Christine Schwab

**Affiliations:** 1 Department of Cognitive Biology, University of Vienna, Vienna, Austria; 2 Haidlhof Research Station, University of Vienna and University of Veterinary Medicine, Bad Vöslau, Austria; 3 Department of Biology and Ecology of Fishes, Leibniz Institute of Freshwater Ecology and Inland Fisheries, Berlin, Germany; 4 Messerli Research Institute, University of Veterinary Medicine Vienna, Medical University of Vienna and University of Vienna, Vienna, Austria; Centre for Ecological and Evolutionary Studies, NETHERLANDS

## Abstract

Consistent individual differences in behaviour, or ‘personality’, are likely to be influenced by development, social context, and species ecology, though few comparative, longitudinal studies exist. Here, we investigated the role of development and social context on personality variation in two identically reared, social corvids: common ravens and carrion crows. We repeatedly presented subjects with a variety of novel food and objects, while alone and in a primarily sibling subgroup, from fledging to sub-adulthood. We predicted that consistent individual differences would emerge later in development, and that conspecific presence would facilitate behavioural similarities. In contrast to our predictions, we found that individuals of both species were highly inconsistent in their behavioural responses throughout the development period. In line with our predictions, though in the ravens only, conspecific presence promoted behavioural similarities as individuals were strongly shaped by their subgroup, and it is likely that these effects were driven by social context rather than relatedness. We discuss these findings in relation to developmental steps and the role of social relations in these species. Overall, our findings highlight that these two species are highly adaptable in their behaviour, and the ravens in particular are strongly influenced by their social environment, which may facilitate cooperation and social learning.

## Introduction

Personality variation refers to the extent of consistent among- and within- individual variation in behaviour [1]. The term ‘personality’ is borrowed from psychology, and in non-human animals, personality refers to behavioural tendencies that differ across individuals though are consistent within individuals over time [2, 3]. These consistent individual differences, i.e. differences among/between individuals, are of interest as they have important implications for ecology and evolution, for instance by impacting learning, performance in cognitive tasks, and on life history strategies [4, 5]. There is now evidence for personality in a wide range of non-human species [6], however, until recently there has been relatively little focus on how personality variation develops across ontogeny [7]. This may be because development reflects change, while the main definitions of animal personality imply stability and consistency in behaviour [8].

In humans, there is a developmental influence on personality, with personality traits becoming increasingly stable with age [9]. However, less is known about the development of personality over the lifetime of non-human animals [7]. The few studies that have investigated this have found mixed results: some species do show pronounced individual personality across ontogeny, or at least between adjacent life stages, such as rats, *Rattus norvegicus* [10], firebugs, *Pyrrhocoris apterus* [11] and field crickets, *Gryllus integer* [12]. Other species show flexibility in their personality development, with some consistency in behavioural traits within life stage, but high within-individual variation between life stages, such as guinea pigs, *Cavia porcellus* [13] and domestic dogs, *Canis familiaris* [14].

There can be differing strengths of personality variation, from highly plastic to highly consistent, though the degree of plasticity in itself has only recently become a target of personality research [15]. The repeatability of behaviour is frequently used to characterize the strength of personality variation; if individuals behave consistently over time (i.e. low within-individual variation), and behave differently from each other (i.e. high among-individual variation), then behaviour is repeatable [1]. Low repeatability therefore implies either high within- and/or low among-individual variation [16]. When low repeatability is caused by high within-individual variation, then individuals could be said to be ‘inconsistent’ in their behaviour. Thus, personality and inconsistency may be considered to represent two ends of the same spectrum, in that personality reflects higher among-individual differences in behaviour, while inconsistency indicates higher within-individual behavioural variation. It is likely that behavioural inconsistency allows individuals to rapidly adjust to their current physical and social environments [8]. Indeed, being able to change behaviour in response to environmental conditions, such as food availability or a novel environment after dispersal, may be adaptive [17]. However, ‘personality’ only exists when the among-individual variation is proportionally larger than within-individual variation to get a statistical signal of differences in average individual behaviour. Therefore inconsistency (i.e. no personality) exists when there is no statistical signal of the individual, i.e. within-individual variation is higher compared to between-individual variation.

Social context can also influence personality, with individuals that closely associate with one another behaving in a similar manner (sparrows, *Passer domesticus* [18]; chimpanzees, *Pan troglodytes*) [19], or showing group-level similarities in personality (common marmosets, *Callithrix jacchus*) [20]. Sociality may promote behavioural similarities, through conformity or social facilitation, such as in Gouldian finches, *Erythrura gouldiae* [20, 21]. Alternatively, sociality may drive behavioural differences through social niche specialization, where repeated interactions with the same group members lead to more pronounced individual differences, such as in social spiders, *Stegodyphus dumicola* [22]. However, despite the likely importance of social environment on personality in social species, personality studies have tended to focus on testing individuals in isolation [21].

Therefore, we explored the development of consistent individual differences, i.e. personality, in both an isolated and social context. We chose two closely related corvid species—common ravens (*Corvus corax*) and carrion/hooded crows (*Corvus corone corone; C*.*c*. *cornix*). These two long-lived, large-brained species have very similar life histories, including being highly social in the nonbreeding stage, with high fission-fusion dynamics across days and months, and being generalists in both diet and habitat use [23–25]. Their developmental steps, including fledging (crows: ~4–5 weeks, ravens ~6 weeks old), independence and dispersal as juveniles (around 6 months old) occur at similar time points, though crows do sexually mature more quickly than ravens (crows: 2–3 years old, ravens: 3–4 years old) [26, 27]. Both species are relatively neophilic, i.e. attracted to novelty, as juveniles, whereas they become highly neophobic, i.e. aversive to novelty, as adults [28, 29]. Early development tends to be a period where individuals are required to cope with novel and changing physical and social environments, as they disperse from their natal territories and join nonbreeding flocks. From late sub-adulthood, the birds tend to be established within nonbreeding flocks and later stable pairs that utilise familiar environments [26, 27].

Our developmental and comparative approach to personality involved repeatedly presenting subjects with different types of novel food and objects, while alone as well as with one or two primarily sibling conspecifics, across the fledgling (1 month old) to sub-adult stage (1.5 years old). In these two species, group-level changes in exploration of novel items across development have been found, with higher exploration generally occurring at the juvenile stage [30]. However, it is possible for group-level differences to occur despite individuals being consistent. For example, if one individual is highly explorative as a juvenile, it may also be highly explorative as a sub-adult, even if the overall group level of exploration increases or decreases. In the present study, our focus was therefore on the development of personality variation, i.e. within- and among- individual variation in behaviour, and the influence of conspecific presence on repeatability. We predicted that, in both species, consistent individual differences would emerge during the later development stages, i.e. repeatability would increase later in life. Due to similarities in life history, feeding ecology and social systems, we did not expect to find overall species differences in personality variation (repeatability in behaviour). However, it is possible that ravens would take longer than crows to develop consistent individual patterns, due to their larger body size and delayed maturation [26]. We quantified repeatability by focussing on temporal and contextual consistency in two behavioural measures–namely frequency of interactions with each novel item type as a measure of an individual’s general exploration/ interest level, and frequency of location changes during tests as a general indicator of an individual’s activity levels.

Social context influences behaviour in both ravens and crows, as presence of conspecifics generally increases exploration behaviour when compared with being alone [30]. However, it is not known whether social context also drives individuals to behave in a similar manner, or if it leads to increased competition that may promote behavioural differences. Stated another way: it is not known whether social context increases or decreases within- and among-individual differences in behaviour, as evidenced by repeatability. Kin relations appear to be important for these birds, as raven siblings spend more time in proximity than non-siblings [31], and juvenile crows feed more readily from novel food after observing their parent do so [32]. Individuals in the present study were tested in primarily sibling subgroups (only 1 raven and 1 crow did not have any siblings), and we expected them to be fairly tolerant of their siblings. We therefore predicted that individuals would consistently behave in a similar way to their subgroup conspecifics, and individuals within subgroups would consistently differ in their behaviour to those in other subgroups, i.e. low within-individual variation and high among-individual variation between subgroups. We did not expect to find species differences with regard to the influence of social context on individual behaviour, as per a previous study [30].

## Materials and Methods

### Subjects

Subjects were nine common ravens (six males, three females) and 10 carrion crows (four males, six females). The ravens were collected from three European zoos at 25–38 days old (fledging ~ 45 days). Four ravens were second generation in captivity, i.e. their grandparents were wild-born, and five ravens were first generation in captivity i.e. their parents were wild-born. The crows were obtained at 10–14 days old (fledging ~ 30 days) from wild nests in Vienna, Austria. The crows tested in this study primarily comprised of carrion crow/hooded crow hybrids, reflecting the overlapping range of the two subspecies across Central Europe [33, 34]. Both species were hand-reared in 2012 under the same conditions, including the same diet, human rearers, enclosure type, and social holding–with their siblings while in the nest, and in a species group post-fledging. Subjects were housed in large, outside aviaries (total size ~ 680 m^2^) at the Haidlhof Research Station, Austria. All testing took place in large, identical test compartments (~ 20m^2^ per compartment), which were directly connected to the main aviaries and contained the same type of substrate and branching. Subjects were trained to be separated individually and in subgroups within these compartments, and participation in tests was voluntary. Subjects were identified individually via coloured leg rings, and were well habituated to people. To control for any differences in individual hunger levels, all subjects were satiated prior to testing. Their daily diet consisted of a variety of meat, fruit, vegetables, yogurt and bread. In general, the subjects were never food-deprived, and water was constantly available.

### Procedure

Subjects were presented with two novel item conditions during separate 10-minute tests: 1. food and 2. small, transferrable objects, representing ‘standard’ personality tests. We were careful to ensure that the birds did not receive any of the experimental food or objects prior to testing to ensure that these items were equally novel to the birds. Both conditions were presented in two different contexts: in the ‘individual’ context while the subject was alone and in temporary visual separation from the group, and in the ‘social’ context while the subject was with one or two primarily sibling conspecifics in temporary visual isolation from the group. Tests were run in the morning on consecutive days with one condition per day. The order of condition and context (individual and social) was counterbalanced across rounds. In the individual context, each condition was repeated ten times per individual, i.e. resulting in a total of 10 rounds, from 1 month old until approx. 1.5 years old (Table 1). Testing started as soon as the birds fledged (left the nest) in May 2012. The first and second rounds took place within the ‘fledgling’ stage (1–2 months old, May-June 2012), the following six rounds, repeated every 3 weeks, within the ‘juvenile’ stage (3–8 months old, July-November 2012), and the final two rounds took place within the ‘sub-adult’ stage (14–18 months old, June and September 2013).

**Table 1:** Overview of testing schedule and experiment items. Individual context tested in all 10 rounds (rounds 1–10) and social context tested in 5 rounds (rounds 1, 4, 7, 8 & 10). / = not tested.

Test round number	Age	Life stage	Individual context		Social context	
			Novel Food	Novel Object	Novel Food	Novel Object
1	1 month old	Fledgling	Mealworms: whole, chilled	Plastic cup: green	Crickets: whole, chilled	Plastic cup: red
2	2 months old	Fledgling	Strawberries: whole	Plastic straw: green	/	/
3	3 months old	Early Juvenile	Cat biscuits: whole, dry	Cardboard juice carton: green	/	/
4	4 months old	Early Juvenile	Kiwi: whole	Plastic Lego block: red	Pineapple: slices	Plastic Lego block: blue
5	5 months old	Early Juvenile	Peanuts: whole	Plastic box: green & purple, butterfly shape	/	/
6	6 months old	Early Juvenile	Corn-on-the-cob: half	Sponge ball: yellow	/	/
7	7 months old	Late Juvenile	Chicks: whole, deceased	Plastic bowling pin: red	Rats: whole, deceased	Plastic bowling pin: green
8	8 months old	Late Juvenile	Oranges: whole	Plastic ball: pink	Khakis: whole	Plastic ball: gold
9	14 months old	Sub-adult	Kohlrabi: whole	Plastic box: blue, rectangle, transparent	/	/
10	18 months old	Sub-adult	Cauliflower: half	Plastic box: green, square, opaque	Peppers: whole	Plastic box: yellow, square, opaque

In the social context, subgroups consisted primarily of same sex siblings, i.e. full siblings from the same clutch—only one raven and one crow did not have any siblings, and two subgroups were mixed sex (one per species). We therefore refer to subgroup composition as consisting of “primarily” sibling birds. Here, each condition was repeated five times per individual from 1 month old until approx. 1.5 years old, with one round at the fledgling stage (1 month old, May 2012), three rounds at the juvenile stage (3–8 months old, July, October and November 2012) and one round at the sub-adult stage (18 months old, September 2013; Table 1).

Between rounds, the types of novel foods and objects presented to the subjects differed, and tests were repeated a minimum of 3 weeks apart, to counter any habituation effects (Table 1). Within each round, between the individual and social context, the novel food and objects also differed, e.g. round 1 in the object condition: individual context—green cup, social context—red cup. Corvids are capable of making simple object feature (including colour) and position discriminations [35], and would therefore perceive these items as novel. Specifically, the novel foods and objects were all similarly sized, small items: food—different types of fruit, vegetables, meat and biscuits, and objects—plastic or wooden items, such as cups, straws, blocks, balls or boxes. In the social context, the same number of food items or objects as subjects were available, i.e. two items when two birds were present, and items were placed on the ground in the centre of a permanent wooden board.

The test began when the item was placed on the ground, and tests were videotaped (Canon HD camera Legria HF510). The 9 ravens were tested in all conditions and test rounds. 10 crows were tested in all conditions and rounds, except the first and last two rounds (round 1 and 2, 9 and 10) when only 8 crows were available for testing, as 2 crows were obtained slightly later than the rest of the group (summer 2012), and 2 crows died (unrelated accident and *Clostridia* infection, summer 2013). Outside of experimental tests, we collected 5-minute focal observations per individual via videotape, while they were in their species groups, 4x per week throughout the entire study period. The observation order of individuals was randomly predetermined prior to each observation session. Observations sessions were evenly dispersed over morning (8-12am) or afternoon (1-6pm) time periods.

### Data Collection and Analysis

Per subject, we recorded two measures throughout the duration of each 10-minute test: frequency of interactions with novel items and frequency of location changes (hereafter ‘frequency’ and ‘activity’). The latter activity measure was taken to quantify individual movements during each test, and comprised of all subject location changes within the test compartment–divided into 4 equal areas and 3 height levels. For the frequency measure, we initially recorded four distinctive behavioural parameters: being in proximity (movement around item within 0.5m^2^), approach (directed movement towards item within 0.5m^2^), touch (less than 3 seconds) and manipulation (more than 3 seconds) with the experiment item. All four frequency parameters (proximity, approach, touch, manipulation) were highly correlated (all intra-class correlation coefficient p<0.05). We therefore calculated a mean value for this measure—average of all four parameters for frequency per subject per test, which we then referred to as frequency of interactions. In general, most individuals did interact with the novel items during the tests (total number of tests interacted with item: food: individual 177/182, social 87/90; object: individual 172/182, social 89/90).

RM coded experiment videos using Solomon Coder (version 14.01.14), with 12% of videos scored by a second coder for inter-rater reliability testing. Spearman’s rank correlations [36] indicated excellent inter-rater reliability (Spearman ρ > 0.991, *p*<0.001).

Statistical analyses comprised linear mixed-effects models [37] using *R* version 3.0.2. We tested for repeatability across rounds in the individual and social context separately. Within each species, we ran a separate linear mixed-effects model for each measure (frequency, activity), using ‘round’ as a fixed factor, and including ‘individual’ as a random effect. We used the resulting variance components to estimate the proportion of variation attributable to differences among individuals, i.e. ‘R’ repeatability [38, 39]. A significant effect of individual was interpreted as evidence that individuals consistently differed, i.e. there was low within- individual variance and high among-individual variance. A significant effect of individual would therefore indicate that individuals consistently differed in X behaviour over Y time period. A significant effect of subgroup would similarly show that the behaviour of subgroups consistently differed over time and between subgroups.

In the social context, we included the additional random factor of ‘subgroup’ (‘individual’ nested within ‘subgroup’) in the linear mixed-effects models. To test for the significance of the random effects (individual and subgroup), we ran a separate model without each random effect and compared the log likelihood ratio (‘L’) of the two models using maximum likelihood. We then also checked whether inclusion of the fixed effect of group size, i.e. if 2 or 3 birds were present, had a significant influence on repeatability by re-running all social context data as above with this fixed factor included in the models (results in S2 Table). Within species, we also tested for individual repeatability between food and object conditions within each round, using the same methods (S3 Table).

Further, we tested if emerging subgroup effects may be driven by social influences or by relatedness, i.e. we tested if subjects behaved in a similar manner as their siblings while alone. To do this, we applied fictitious subgroups to the individual context data (tested while alone), by assigning individuals to the same subgroups as they were tested in the social context. For example, individual 1, 2 and 3 were tested together in the social context in subgroup ‘A’–so for these analyses of the individual context data, we grouped the data from these individuals (while tested alone) together under subgroup A. We then repeated the same tests that were run previously on the social context data.

In the individual and social contexts, we tested across rounds: i) rounds 1–10 to represent overall repeatability from fledgling to sub-adult (individual: all 10 rounds; social: all 5 rounds: 1, 4, 7, 8, 10), ii) rounds 1–6 (individual)/ rounds 1–4 (social context) for early life repeatability from fledgling to early juvenile, and iii) rounds 7–10 for later life repeatability from late juvenile to sub-adult (individual: all 4 rounds; social: 3 rounds: 7, 8 and 10) (Table 1). Figures were produced using SPSS version 19 and Adobe Illustrator. The supporting data set can be accessed on Figshare: http://dx.doi.org/10.6084/m9.figshare.1529828

Additionally, due to the unexpected species differences found, we explored affiliative relations within i) sibling groups, and ii) tested subgroups, for both species. We tested both i) and ii) as these subgroup categories differed in composition (Table 2). Specifically, for the ravens, the i) sibling group model comprised of 9 ravens in 3 sibling subgroups—1 four-bird, 1 three-bird, 1 two-bird, and the ii) tested subgroup model comprised of 9 ravens in 4 primarily sibling subgroups—1 three-bird, 3 two-bird, as the four-bird subgroup was split into 2 two-bird subgroups, and 1 two-bird subgroup contained the 1 raven with no siblings (‘Rufus’). For the crows, the i) sibling group model comprised of 9 crows in 3 sibling subgroups—1 four-bird, 1 three-bird, 1 two-bird, and the ii) tested subgroup model comprised of 10 crows in 4 primarily sibling subgroups—2 three-bird, 2 two-bird, as the four-bird sibling group was split into 1 two-bird and 1 three-bird subgroups–the latter containing the 1 crow with no siblings (‘Corbie’). For ii) individuals were assigned to tested subgroups by relatedness and sex where possible. To determine affiliation between individuals, we recorded frequencies of contact sitting (two birds sitting next to each other within one body length), obtained via 5-minute focal observations recorded from May (crows)/ June (ravens) 2012 to September 2013. Inter-rater reliability between the focal observation video coders was very high (Cohen’s kappa κ = 0.84, p = <0.001). By using constant homophily models (Ucinet 6.365), we tested the hypothesis whether subgroups have a preference for in-group ties compared to out-group ties, i.e. if they preferentially contact sit more frequently with individuals that are i) siblings over non-siblings and ii) from within their tested subgroup over those outside this subgroup.

**Table 2:** Sibling group and tested subgroups composition in both species. All test participation was voluntary for the birds, and 1 available male raven (Max) did not participate in tests, though was included in the sibling group analyses as he was a sibling (to Paul). F = female, M = male.

Ravens				Crows			
Subject	Sex	Tested subgroup	Sibling group	Subject	Sex	Tested subgroup	Sibling group
Adele	F	A	A	Corbie	M	A	No sibling
Laggie	M	A	A	Saul	M	A	A
Tom	M	A	A	Signore	M	A	A
George	M	B	B	Daisy	F	B	B
Horst	M	B	B	Emily	F	B	B
Louise	F	C	B	Suki	F	B	B
Nobel	F	C	B	Juno	F	C	C
Paul	M	D	C	Lilith	M	C	C
Rufus	M	D	No sibling	Peppi	F	D	A
Max	M	Not tested	C	Rainer	F	D	A

### Ethics Statement

We received permission to remove crow nestlings from the wild by the Magistrate of Vienna for Environmental Protection, application number MA 22-355/2012/4. Permission to keep animals at Haidlhof Research Station was obtained from the Austrian Ministry for Science and Research, license number BMWFW-66.006/0011-WF/II3b/2014 from 22 May 2014. All birds were housed in accordance with Austrian Law and local government guidelines. This study was entirely non-invasive, and therefore not classified as an animal experiment according to Austrian Law (§2. Federal Law Gazette No.501/1989). The study was reviewed and approved by the Internal Animal Welfare Board at the Faculty of Life Sciences, University of Vienna (2014.012).

## Results

### Temporal repeatability in the individual context: inconsistency in individual behaviour when alone

We tested for repeatability in an individual setting across the entire developmental period from fledgling to sub-adult (rounds 1–10), as well as from fledgling to early juvenile (rounds 1–6), and late juvenile to sub-adult (rounds 7–10) to represent early and late development respectively. There was little evidence for strong consistent individual differences in behaviour as we found high within- and low among- individual variation in both species across the development period, as well as during the early and late development stages separately. Specifically, we found that individuals did not consistently differ in frequency or activity in the food and object conditions over rounds 1–10, 1–6 or 7–10 (Repeatability range: R<0.001–0.18; S1 Table).

### Contextual repeatability: inconsistency in individual behaviour between conditions

For both species, we also tested for repeatability between the food and object conditions within each round, for both the individual and social context. We found that there were few significant effects of individual between conditions for either species (S3 Table). While alone, raven individuals consistently differed in their frequency of interactions with the food versus objects at round 7 and 10 (late development; round 7: R = 0.259, l = 4.02, *p* = 0.045; round 10: R = 0.825, L = 10.008, *p* = 0.002). They also consistently differed in their activity levels towards food and objects in round 4 (early development) (R = 0.201, L = 28.9, *p* = 0.001). However, raven individuals did not show consistent individual differences in the social context (S3 Table). While alone, crow individuals consistently differed in frequency of interactions with food versus objects at round 7 (late development; R = 0.336, l = 0.434, *p* = 0.037). In the social context, they also consistently differed in their activity levels in response to food versus objects at round 8 (late development; R = 0.593, L = 2.14, *p* = 0.042).

### Temporal repeatability in the social context: ravens exhibit individual differences in a subgroup

**Fig 1:**
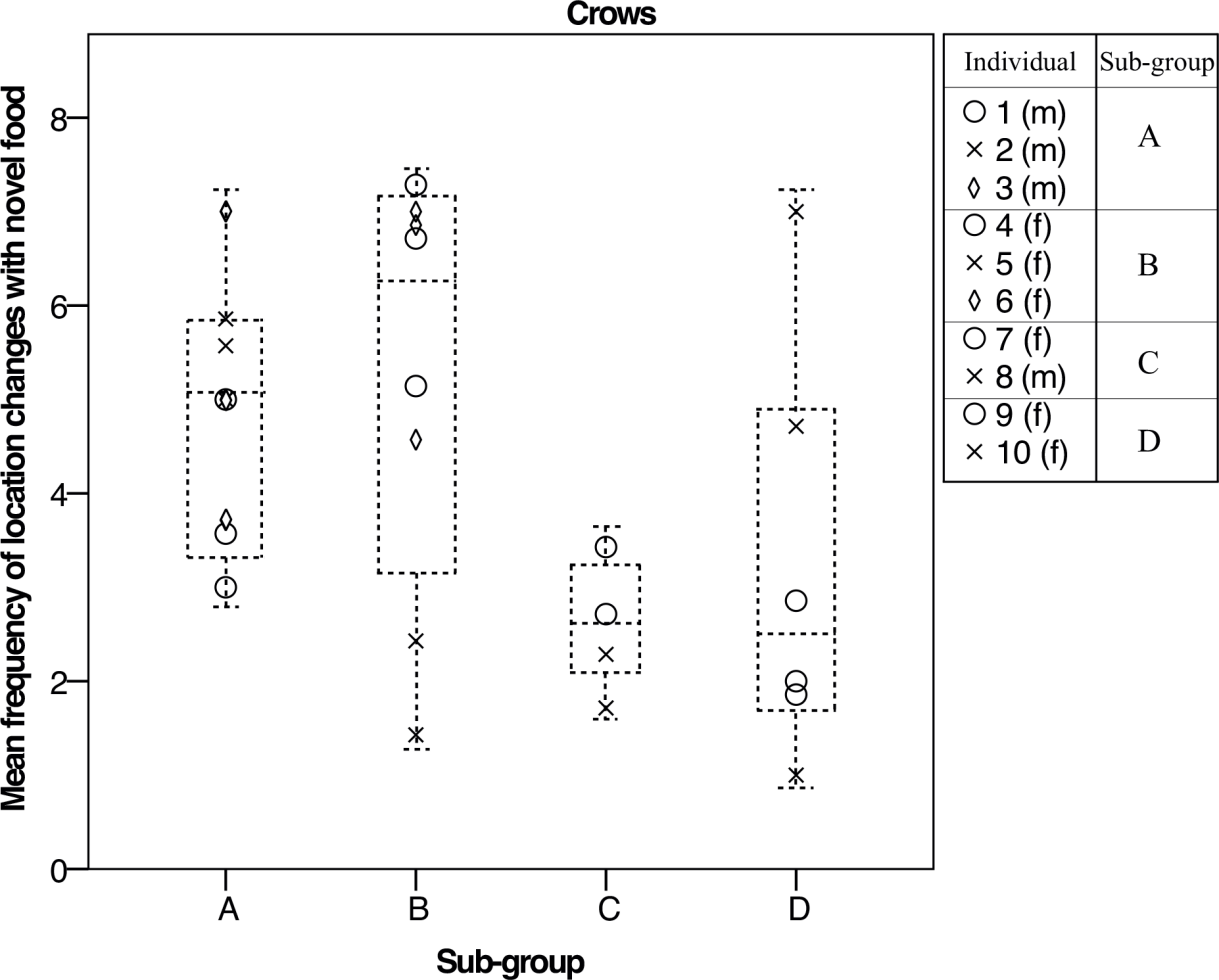
Mean activity in novel food condition across late development in social context for crows. We found that individuals differed consistently in behaviour (frequency of location changes) across late development (rounds 7, 8 and 10). f = female, m = male.

We tested for repeatability while in the social context across the entire developmental period from fledgling to sub-adult (rounds 1–10), as well as from fledgling to early juvenile (rounds 1–4), and late juvenile to sub-adult (rounds 7–10) to represent early and late development respectively. Overall, in the presence of one or more conspecifics, we found some significant individual effects, i.e. low within- individual variance and high among-individual variance, in both species, but in later development only (Table 3 and S2 Table). In the crows, individuals consistently differed in activity levels in response to novel food over late development only (round 7–10: activity: R = 0.5, L = 4.7, *p* = 0.029; Fig 1). There were no significant effects of subgroup; hence subgroups did not consistently differ in behaviour over time or from one another (Table 3).

**Table 3:** Significant repeatability over time in social context for novel food and object conditions for both species. In the ravens, individuals and subgroups consistently differed in their responses to food and objects, particularly over the late development stages. In the crows, individuals did not generally differ consistently in their behaviour over time. R = repeatability, L = likelihood ratio. Significant results given in bold. Full test results in S2 Table.

Species	Rounds	Measure	Individual/ subgroup effect	Novel Food	Novel Object
Raven	1–10	Frequency	Subgroup	**R = 0.18, L = 3.9, *p* = 0.047**	R = 0.053, L = 0.46, *p* = 0.49
		Activity	Subgroup	**R = 0.27, L = 7.3, *p* = 0.007**	R = 0.94, L = 0.27, *p*>0.999
Raven	7–10	Frequency	Individual	R = 0.045, L = 0.09, *p* = 0.76	**R = 0.49, L = 5.5, *p* = 0.019**
		Frequency	Subgroup	**R = 0.32, L = 4.4, *p* = 0.035**	**R = 0.59, L = 13.4, *p*<0.001**
	7–10	Activity	Individual	**R = 0.52, L = 6.1, *p* = 0.014**	R = 0.1, L = 0.25, *p* = 0.617
		Activity	Subgroup	**R = 0.57, L = 13, *p*<0.001**	R = 0.2, L = 2.08, *p* = 0.148
Crow	7–10	Activity	Individual	**R = 0.5, L = 4.7, *p* = 0.029**	R<0.001, L<0.001, *p*>0.999

**Fig 2:**
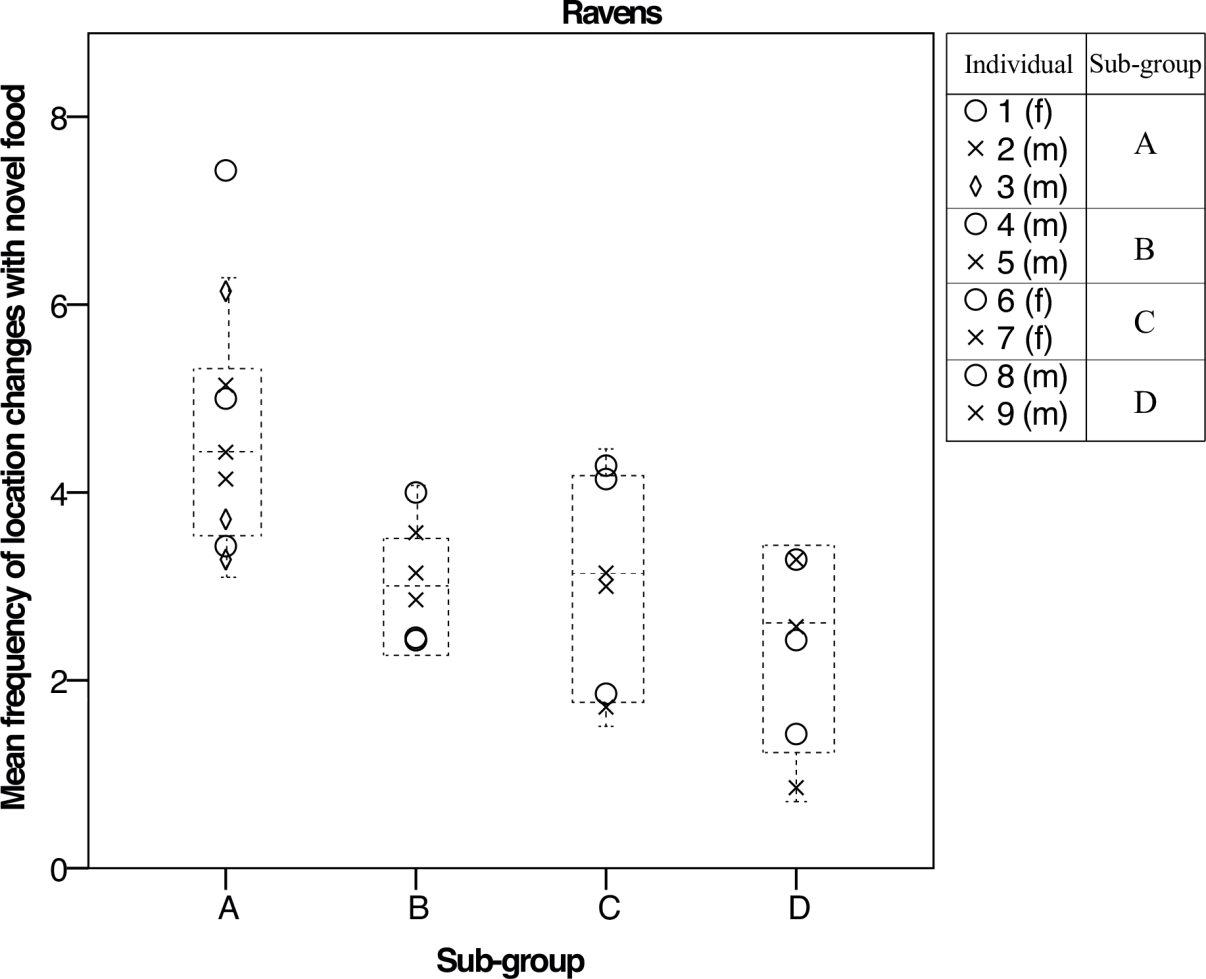
Mean activity in novel food condition across late development in social context for ravens. We found that individuals and subgroups differed consistently in behaviour (frequency of location changes) across late development (rounds 7, 8 and 10). f = female, m = male.

In the ravens, we found that individuals consistently differed over late development in the novel food and object conditions (round 7–10: food: activity: R = 0.52, L = 6.1, *p* = 0.014; object: frequency: R = 0.49, L = 5.5, *p* = 0.019). In the ravens, we also found that subgroups consistently differed in responses to food and objects, with differences among subgroups being stronger than differences within subgroups. In the novel food condition, subgroups consistently differed in both frequency and activity measures over the entire developmental period, as well as across late development (round 1–10: frequency: R = 0.18, L = 3.9, *p* = 0.047; activity: R = 0.27, L = 7.3, *p* = 0.007; round 7–10: frequency: R = 0.32, L = 4.4, *p* = 0.035; activity: R = 0.57, L = 13, *p*<0.001; Fig 2; Table 3). In the novel object condition, raven subgroups consistently differed in the frequency measure across late development (round 7–10: frequency: R = 0.59, L = 13.4, *p*<0.001). We therefore found that both individual and subgroups consistently differed in behaviour towards novel food and objects across late development–the late juvenile to sub-adult stages. These results show that, in the ravens only, there was a strong influence of subgroup on individual behaviour, indicating the social context decreases within-individual variation and increases among-individual variation between the subgroups. Additionally, we found no significant effect of group size in either species (S2 Table).

### Social context or relatedness: ravens do not behave similarly to subgroup conspecifics while alone

We analysed the individual results from the solitary condition as if the subjects were in subgroups, in order to explore whether subjects behaved similarly to their tested subgroup conspecifics, which were primarily siblings, regardless of their physical presence. We found that individuals and these fictional subgroups did not consistently differ in behaviour towards food or objects (S4 Table), indicating that subjects did not behave in a similar manner to their tested subgroup conspecifics while alone.

### Sibling relationship quality: affiliation rather than relatedness

Using the affiliation data obtained through focal observations, we found that both species showed significant affiliative, i.e. contact sit, preferences for siblings over non-siblings in general in the (i) within sibling group comparison (raven: R^2^ = 0.335, *p* = 0.0041; crow: R^2^ = 0.191, *p* = 0.0087) (S5 Table). However, only the ravens showed significant affiliative preferences for individuals within their tested subgroup over other subgroups in the (ii) within tested subgroup comparison (raven: R^2^ = 0.299, *p* = 0.0027; crow: R^2^ = 0.023, *p* = 0.281).

## Discussion

We took an integrative approach to investigate personality variation in common ravens and carrion crows during development, while alone as well as with one or more primarily sibling conspecifics. We found that, in general, individuals of both species were highly inconsistent, i.e. they showed low repeatability, in their behaviour while alone and in a social context across the entire development period. These findings, in addition to a lack of contextual repeatability, indicated that there is little evidence for strong consistent individual differences in either species from fledgling to the sub-adult stage (1.5 years old). Rather, the birds displayed high inconsistency in their behavioural responses to novelty during development.

In the ravens though unexpectedly not in the crows, subgroups consistently differed in responses to novel food from early on in their development, and across the later life stages for novel objects—indicating subgroup-level similarities in behaviour. In two situations, both individual and subgroups consistently differed in behaviour—namely activity in the novel food condition, and frequency of interactions with the item in the novel object condition. This finding indicates that, in the ravens only, differences among subgroups were stronger than differences within subgroups, and then individuals within those subgroups also exhibited consistent individual differences (low within- individual variance and high among-individual variation). Similar to our findings, a recent study in marmosets found that individuals were highly flexible in personality, though they showed group-level similarity with their family members in their personality traits–referred to as ‘group-personality’ [20]. Group-personality is likely to be highly adaptive for promoting cooperation in social species, and may also occur as a by-product of social learning [20].

Behavioural similarities within kin subgroups may be driven by genetic influences as opposed to the social context. As we found no individual or subgroup effects on the individual context data, i.e. while alone, it is therefore likely that the behavioural similarities that we found in the ravens were driven by the social context rather than kin similarities, which may indicate short-term social effects, such as social facilitation, on highly flexible traits [20]. Both common ravens and carrion crows show cooperative behaviours and social learning [31, 40]. For instance, ravens will recruit conspecifics to foraging sites [41], and a Spanish carrion crow population shows cooperative breeding [42]. These findings, in addition to having similar social systems, make the apparent species differences in group-personality found in the present study unexpected.

It is possible that sibling relations play a greater role in raven interactions during development than in crow interactions. Previous studies have highlighted the importance of kin relations in ravens, with individuals showing more valuable relationships between kin than non-kin [43] and enhanced social learning between siblings [31]. Direct comparisons between common ravens and carrion crows are lacking. However, we did a post-hoc investigation of preferences for sibling versus non-sibling, and within tested subgroup versus other subgroups, in both species using focal observations collected throughout the study period. We found that sibling relations were important for both species. However, affiliation within the tested subgroups was more pronounced for the ravens than the crows. We therefore argue that the behavioural similarities among the raven subgroups were being driven by higher patterns of affiliation among the subgroup members, compared to the crows. The apparent species difference could thus be due to the selection of the subgroups in our experiment and/or the very relationships formed in our study population. It is possible therefore that individual crows may still behave in a similar manner to their conspecifics during development, depending on their affiliation with those individuals. As our subgroups differed in composition (ravens: 3 two-bird and 1 three-bird subgroups, crows: 2 two-bird and 2 three-bird subgroups), we tested for an effect of group size, and found no group size effect in the ravens or crows.

Regardless of the developmental stages selected, we found individuals in both species did not consistently differ in responses to novel items when tested alone, indicating low repeatability throughout development. There are several potential explanations for this finding. Low repeatability may be related to individuals experiencing differential selection over time, and selection favours plasticity and flexibility [44]. During development, wild ravens and crows will experience changing physical and social environmental conditions, due to dispersal and their high fission-fusion social systems [26, 27]. Although the environment is likely to be more stable in captivity, in terms of access to food and conspecifics, social experiences and interactions with kin and non-kin will still have differed between individuals in our study in a similar manner to the field. Flexibility in the behaviour of these species may therefore be expected to be adaptive during their development.

As we used two conditions that both required interaction with a novel item (food, object), it could be suggested that, although these birds don’t show strong individual differences in this type of context, they may do so in others. This is possible, however, our finding that there was also little evidence for contextual repeatability within rounds (i.e. repeatability between conditions), indicates that the individuals may be responding to the two conditions in a different manner. The conditions may lead to functionally different behaviours, such as presenting a differing degree of risk; for instance, interacting with a novel object may constitute less risk than a novel food, which could be toxic, or alternatively, an object may be of less interest than food.

Further, in humans, behavioural consistency increases with maturity [9], and in dumpling squid, *Euprymna tasmanica*, major behavioural reorganization was found around the time of sexual maturity, with higher consistency in behaviour prior to and following maturation [45]. The birds in the present study were not yet sexually mature, and so it would be worthy to explore personality in adult birds, though it would be difficult to further explore the role of kin as adults are typically held in breeding pairs. Despite this, our findings that group-living individuals that were identically reared and housed were remarkably inconsistent in their behaviour, and in particular, that the ravens only, despite becoming sexually mature later than the crows, were constrained by the presence of their kin subgroup from early on, renders these potential limitations unlikely.

We therefore present the first comparative study of personality variation in common ravens and carrion crows across ontogeny, and, in general, found both species to be remarkably inconsistent in their individual behaviour while alone and in the social context. The ravens did show subgroup similarities, though subgroups consistently differed from one another, resulting in a ‘group-personality’, which was present from early on, and may serve to enhance cooperative behaviours and social learning. These findings warrant further exploration, particularly as to the role of kin and affiliation during development in both species, and in the meantime, contribute to expanding our understanding of personality variation across development in social, long-lived non-human species.

## Supporting Information

S1 Table Repeatability over time in individual context (while alone) for novel food and object conditions for both species. (PDF)

S2 Table Repeatability over time in social context for novel food and object conditions for both species. (PDF)

S3 Table Repeatability between novel food and object conditions in individual and social context for both species. (PDF)

S4 Table Repeatability over time in individual context: testing whether, in the ravens, subgroup effects arose from social context or similarity in behaviour to kin. (PDF)

S5 Table Constant homophily models testing for in-group preferences of affiliative, i.e. contact sit interactions in a) sibling groups and b) tested subgroups in ravens and crows. (PDF)

## References

[1] Bell AM, Hankison SJ, Laskowski KL. The repeatability of behaviour: a meta-analysis. Anim Behav. 2009;77: 771–83. 24707058 10.1016/j.anbehav.2008.12.022 PMC3972767

[2] Caspi A, Roberts BW, Shiner RL. Personality development: stability and change. Annu Rev Psychol. 2005;56: 453–84. 15709943 10.1146/annurev.psych.55.090902.141913

[3] Reale D, Reader SM, Sol D, McDougall PT, Dingemanse NJ. Integrating animal temperament within ecology and evolution. Biol Rev Camb Philos. 2007;82: 291–318. 10.1111/j.1469-185X.2007.00010.x 17437562

[4] Sih A, Del Giudice M. Linking behavioural syndromes and cognition: a behavioural ecology perspective. Phil T R Soc B. 2012;367: 2762–72. 10.1098/rstb.2012.0216 PMC3427552 22927575

[5] Réale D, Dingemanse NJ. Animal Personality. Chichester: eLS John Wiley & Sons, Ltd; 2001.

[6] Gosling SD, John OP. Personality dimensions in nonhuman animals: a cross-species review. Curr Dir Psychol Sci. 1999;8: 69–75.

[7] Stamps J, Groothuis TG. The development of animal personality: relevance, concepts and perspectives. Biol Rev Camb Philos. 2010;85: 301–25. 10.1111/j.1469-185X.2009.00103.x 19961473

[8] Groothuis TG, Trillmich F. Unfolding personalities: the importance of studying ontogeny. Dev Psychobiol. 2011;53: 641–55. 10.1002/dev.20574 21866544

[9] Roberts BW, DelVecchio WF. The rank-order consistency of personality traits from childhood to old age: a quantitative review of longitudinal studies. Psychol Bull. 2000;126: 3–25. 10668348 10.1037/0033-2909.126.1.3

[10] Rodel HG, Meyer S. Early development influences ontogeny of personality types in young laboratory rats. Dev Psychobiol. 2011;53: 601–13. 10.1002/dev.20522 21866542

[11] Gyuris E, Feró O, Barta Z. Personality traits across ontogeny in firebugs, *Pyrrhocoris apterus*. Anim Behav. 2012;84: 103–9.

[12] Niemelä P, Vainikka A, Lahdenperä S, Kortet R. Nymphal density, behavioral development, and life history in a field cricket. Behav Ecol Sociobiol. 2012;66: 645–52.

[13] Guenther A, Finkemeier MA, Trillmich F. The ontogeny of personality in the wild guinea pig. Anim Behav. 2014;90: 131–9.

[14] Riemer S, Müller C, Virányi Z, Huber L, Range F. Validity of ealy behavioral assessments in dogs–A longitudinal study. J Vet Behav. 2014;9: e10–e1. 10.1371/journal.pone.0101237 PMC4086890 25003341

[15] Dingemanse NJ, Wolf M. Between-individual differences in behavioural plasticity within populations: causes and consequences. Anim Behav. 2013;85: 1031–9.

[16] Gabor CR, Aspbury AS. Non-repeatable mate choice by male sailfin mollies, Poecilia latipinna, in a unisexual-bisexual mating complex. Behav Ecol. 2008;19: 871–8.

[17] Stamps JA, Groothuis TG. Developmental perspectives on personality: implications for ecological and evolutionary studies of individual differences. Phil Trans R Soc B. 2010;365: 4029–41. 10.1098/rstb.2010.0218 21078655 PMC2992751

[18] Templeton CN, Reed VA, Campbell SE, Beecher MD. Spatial movements and social networks in juvenile male song sparrows. Behav Ecol. 2011;23: 141–52. 22479140 10.1093/beheco/arr167 PMC3242974

[19] Massen JJM, Koski SE. Chimps of a feather sit together: chimpanzee friendships are based on homophily in personality. Evol Hum Behav. 2014;35: 1–8.

[20] Koski SE, Burkart JM. Common marmosets show social plasticity and group-level similarity in personality. Sci Rep. 2015;5: 8878 10.1038/srep08878 25743581 PMC5155412

[21] King AJ, Williams LJ, Mettke-Hofmann C. The effects of social conformity on Gouldian finch personality. Anim Behav. 2015;99: 25–31.

[22] Modlmeier AP, Laskowski KL, DeMarco AE, Coleman A, Zhao K, Brittingham HA, et al Persistent social interactions beget more pronounced personalities in a desert-dwelling social spider. Biol Lett. 2014;10: 20140419 10.1098/rsbl.2014.0419 25165452 PMC4155910

[23] Goodwin D. Crows of the World. Ithaca NY: Cornell University Press; 1976.

[24] Poole AF, Gill FB. The Birds of North America: Life Histories for the 21st Century. Philadelphia: Birds of North America Inc; 2002.

[25] Braun A, Walsdorff T, Fraser O, Bugnyar T. Socialized sub-groups in a temporary stable Raven flock? J Ornithol. 2012;153: 97–104. 25892747 10.1007/s10336-011-0810-2 PMC4398859

[26] Snow DW, Perrins CM, Gillmor R. The Birds of the Western Palearctic: Passerines. Oxford: Oxford University Press; 1998.

[27] Ratcliffe D, Rose C. The Raven. London: Bloomsbury Publishing; 2010.

[28] Heinrich B. Neophilia and exploration in juvenile common ravens, Corvus corax. Anim Behav. 1995;50: 695–704.

[29] Kilham L. The American Crow and the Common Raven. College Station: Texas A & M University Press; 1991.

[30] Miller R, Bugnyar T, Pölzl K, Schwab C. Differences in exploration behaviour in common ravens and carrion crows during development and across social context. Behav Ecol Sociobiol. 2015;69: 1209–20. 26097282 10.1007/s00265-015-1935-8 PMC4464646

[31] Schwab C, Bugnyar T, Schloegl C, Kotrschal K. Enhanced social learning between siblings in common ravens, Corvus corax. Anim Behav. 2008;75: 501–8. 25948875 10.1016/j.anbehav.2007.06.006 PMC4417712

[32] Chiarati E, Canestrari D, Vera R, Baglione V. Subordinates benefit from exploratory dominants: response to novel food in cooperatively breeding carrion crows. Anim Behav. 2012;83: 103–9.

[33] Glutz von Blotzheim U. Handbuch der Vögel Mitteleuropas. Wiesbaden: Aula-Verlag; 1993.

[34] de Knijff P. How carrion and hooded crows defeat Linnaeus's curse. Science. 2014;344: 1345–6. 10.1126/science.1255744 24948724

[35] Range F, Bugnyar T, Kotrschal K. The performance of ravens on simple discrimination tasks: a preliminary study. Acta Ethologica. 2008;11: 34–41. 25948877 10.1007/s10211-008-0039-0 PMC4417711

[36] Martin P, Bateson P. Measuring Behaviour: An Introductory Guide. Cambridge: Cambridge University Press; 2007.

[37] Baayen RH. Analyzing Linguistic Data: A Practical Introduction to Statistics using R. Cambridge: Cambridge University Press; 2008.

[38] Lessells CM, Boag PT. Unrepeatable repeatabilities: a common mistake. Auk. 1987;104N: 116–21.

[39] Nakagawa S, Schielzeth H. Repeatability for gaussian and non-gaussian data: a practical guide for biologists. Biol Rev Camb Philos 2010;85: 935–56. 10.1111/j.1469-185X.2010.00141.x 20569253

[40] Miller R, Schiestl M, Whiten A, Schwab C, Bugnyar T. Tolerance and social facilitation in the foraging behaviour of free-ranging crows (*Corvus corone corone; C*. *c*. *cornix*). Ethology. 2014;120: 1248–55. 25937686 10.1111/eth.12298 PMC4415146

[41] Heinrich B, Marzluff JM. Do common ravens yell because they want to attract others? Behav Ecol Sociobiol. 1991;28: 13–21.

[42] Baglione V, Canestrari D, Marcos J, Ekman J. Kin selection in cooperative alliances of carrion crows. Science. 2003;300: 1947–9. 12817149 10.1126/science.1082429

[43] Fraser ON, Bugnyar T. The quality of social relationships in ravens. Anim Behav. 2010;79: 927–33. 25821236 10.1016/j.anbehav.2010.01.008 PMC4373546

[44] Westneat D, Fox CW. Evolutionary Behavioral Ecology. Oxford: Oxford University Press; 2010.

[45] Sinn DL, Gosling SD, Moltschaniwskyj NA. Development of shy/bold behaviour in squid: context-specific phenotypes associated with developmental plasticity. Anim Behav. 2008;75: 433–42.

