# Supplementary material for: Socially Driven Consistent Behavioural Differences during Development in Common Ravens and Carrion Crows
Source: PLoS One. 2016 Feb 5;11(2):e0148822. doi: 10.1371/journal.pone.0148822 (PMC4746062; doi:10.1371/journal.pone.0148822)
Supplement: S1 Table — (PDF) [file pone.0148822.s001.pdf]

S1 Table. Repeatability over time in individual context (while alone) for novel food and object conditions for both species. There were no significant effects of individual; therefore individuals did not consistently differ in responses to food or objects over time. Individual context tested in ten rounds (rounds 1-10; fledging to sub-adult stage). Measures: frequency of interaction with test item, activity – frequency of location changes. R= repeatability, L = likelihood ratio

**Species**	**Rounds**	**Measure**	**Novel Food**	**Novel Object**
**Raven**	1-6	Frequency	R=0.013, L=0.02, *p*=0.88	R=0.107, L=1.22, *p*=0.268
		Activity	R=0.14, L=1.75, *p*=0.18	R<0.001, L<0.001, *p*>0.999
	1-10	Frequency	R<0.001, L<0.001, *p*>0.999	R<0.001, L<0.001, *p*>0.999
		Activity	R=0.04, L=0.65, *p*=0.42	R<0.001, L<0.001, *p*>0.999
	7-10	Frequency	R=0.045, L=0.09, *p*=0.76	R<0.001, L<0.001, *p*>0.999
		Activity	R=0.093, L=0.039, *p*=0.53	R<0.001, L<0.001, *p*>0.999
**Crow**	1-6	Frequency	R<0.001, L<0.001, *p*>0.999	R=0.04, L=0.19, *p*=0.65
		Activity	R=0.18, L=3.48, *p*=0.062	R=0.06, L=0.43, *p*=0.51
	1-10	Frequency	R<0.001, L<0.001, *p*>0.999	R=0.094, L=2.46, *p*=0.116
		Activity	R=0.12, L=3.52, *p*=0.06	R=0.009, L=0.036, *p*=0.84
	7-10	Frequency	R=0.12, L=0.58, *p*=0.45	R=0.095, L=0.41, *p*=0.52
		Activity	R=0.18, L=1.13, *p*=0.29	R<0.001, L<0.001, *p*>0.999
