# Supplementary material for: Socially Driven Consistent Behavioural Differences during Development in Common Ravens and Carrion Crows
Source: PLoS One. 2016 Feb 5;11(2):e0148822. doi: 10.1371/journal.pone.0148822 (PMC4746062; doi:10.1371/journal.pone.0148822)
Supplement: S2 Table — (PDF) [file pone.0148822.s002.pdf]

S2 Table. Repeatability over time in social context for novel food and object conditions for both species. We found that individuals in both species, and subgroups in the ravens only, differed consistently in behaviour towards novel food and objects across late development. Group size was included separately as a fixed effect (i.e. if 2 or 3 birds were present), with no significant effect found in either species. Social context tested in five rounds (rounds 1, 4, 7, 8 and 10; fledging to sub-adult stage). R= repeatability, L = likelihood ratio. Significant results given in bold.

**Species**	**Rounds**	**Measure**	**Individual/ subgroup effect**	**Novel Food**	**Novel Object**
**Raven**	1, 4	Frequency	Individual	R=0.215, L=0.39, *p*=0.532	R<0.001, L<0.001, *p*>0.999
			Subgroup	R=0.175, L=0.29, *p*=0.59	R=0.08, L=0.182, *p*=0.67
		Activity	Individual	R=0.0097, L=0.00076, *p*=0.978	R=0.138, L=0.151, *p*=0.698
			Subgroup	R=0.188, L=0.836, *p*=0.361	R<0.001, L<0.001, *p*>0.999
	1-10	Frequency	Individual	R=0.12, L=1.04, *p*=0.308	R<0.001, L<0.001, *p*>0.999
			Subgroup	**R=0.18, L=3.9, *p*=0.047** (group size: R<0.001, L<0.001, *p*>0.999)	R=0.053, L=0.46, *p*=0.49
		Activity	Individual	R=0.17, L=1.9, *p*=0.164	R<0.001, L<0.001, *p*>0.999
			Subgroup	**R=0.27, L=7.3, *p*=0.007** (group size: R<0.001, L<0.001, *p*>0.999)	R=0.094, L=2.7, *p*>0.999
	7-10	Frequency	Individual	R=0.045, L=0.09, *p*=0.76	**R=0.49, L=5.5, *p*=0.019** (group size: R=0.059, L=0.076, *p*=0.783)
			Subgroup	**R=0.32, L=4.4, *p*=0.035 (**group size: R=0.04, L=0.054, *p*=0.816)	**R=0.59, L=13.4, *p*<0.001 (**group size: R=0.33, L=2.56, *p*=0.102)
		Activity	Individual	**R=0.52, L=6.1, *p*=0.014 (**group size: R<0.001, L<0.001, *p*>0.999)	R=0.1, L=0.25, *p*=0.617
			Subgroup	**R=0.57, L=13, *p*<0.001 (**group size: R=0.07, L=0.14, *p*=0.7)	R=0.2, L=2.08, *p*=0.148
**Crow**	1, 4	Frequency	Individual	R=0.77, L=3.488, *p*=0.0618	R=0.199, L=0.247, *p*=0.62
			Subgroup	R<0.001, L<0.001, *p*>0.999	R<0.001, L<0.001, *p*>0.999
		Activity	Individual	R<0.001, L<0.001, *p*>0.999	R<0.001, L<0.001, *p*>0.999
			Subgroup	R=0.419, L=2.059, *p*=0.151	R<0.001, L<0.001, *p*>0.999
	1-10	Frequency	Individual	R<0.001, L<0.001, *p*>0.999	R<0.001, L<0.001, *p*>0.999
			Subgroup	R=0.054, L=0.495, *p*=0.48	R<0.001, L<0.001, *p*>0.999
		Activity	Individual	R=0.204, L=1.85, *p*=0.17	R<0.001, L<0.001, *p*>0.999
			Subgroup	R-0.149, L=1.388, *p*=0.238	R=0.11, L=1.06, *p*=0.3
	7-10	Frequency	Individual	R<0.001, L<0.001, *p*>0.999	R<0.001, L<0.001, *p*>0.999
			Subgroup	R=0.085, L=0.45, *p*=0.5	R<0.001, L<0.001, *p*>0.999
		Activity	Individual	**R=0.5, L=4.7, *p*=0.029 (**group size: R=0.36, l=1.95, *p*=0.163)	R<0.001, L<0.001, *p*>0.999
			Subgroup	R=0.219, L=1.54, *p*=0.21	R=0.23, L=1.5, *p*=0.22
