# Supplementary material for: Socially Driven Consistent Behavioural Differences during Development in Common Ravens and Carrion Crows
Source: PLoS One. 2016 Feb 5;11(2):e0148822. doi: 10.1371/journal.pone.0148822 (PMC4746062; doi:10.1371/journal.pone.0148822)
Supplement: S3 Table — (PDF) [file pone.0148822.s003.pdf]

S3 Table. Repeatability between novel food and object conditions in individual and social context for both species. In general, individuals did not behave consistently towards food and objects within each round. Individual context tested in rounds 1-10, social context tested in rounds 1, 4, 7, 8 and 10. R= repeatability, L = likelihood ratio. Significant results given in bold.

Species	Round	Measure	Context	Food, object
Raven	1	Frequency	Individual	R=0.45, L=1.965, *p*=0.161
			Social	R<0.001, L<0.001, *p*>0.999
		Activity	Individual	R=0.154, L=0.202, *p*=0.653
			Social	R=0.586, l=3.646, *p*=0.0562
	2	Frequency	Individual	R=0.483, L=2.28, *p*=0.132
		Activity	Individual	R<0.001, L<0.001, *p*>0.999
	3	Frequency	Individual	R<0.001, L<0.001, *p*>0.999
		Activity	Individual	R=0.529, L=2.82, *p*=0.0926
	4	Frequency	Individual	R=0.084, L=0.027, *p*=0.87
			Social	R<0.001, L<0.001, *p*>0.999
		Activity	Individual	**R=0.201, L=28.9, *p*=0.001**
			Social	R<0.001, L<0.001, *p*>0.999
	5	Frequency	Individual	R<0.001, L<0.001, *p*>0.999
		Activity	Individual	R=0.11, L=0.034, *p*=0.853
	6	Frequency	Individual	R<0.001, L<0.001, *p*>0.999
		Activity	Individual	R<0.001, L<0.001, *p*>0.999
	7	Frequency	Individual	**R=0.259, l=4.02, *p*=0.045**
			Social	R<0.001, L<0.001, *p*>0.999
		Activity	Individual	R<0.001, L<0.001, *p*>0.999
			Social	R=0.166, L=0.238, *p*=0.626
	8	Frequency	Individual	R=0.116, L=0.115, *p*=0.734
			Social	R<0.001, L<0.001, *p*>0.999
		Activity	Individual	R=0.06, L=0.04, *p*=0.86
			Social	R=0.102, L=0.009, *p*=0.762
	9	Frequency	Individual	R<0.001, L<0.001, *p*>0.999
		Activity	Individual	R<0.001, L<0.001, *p*>0.999
	10	Frequency	Individual	**R=0.825, L=10.008, *p*=0.002**
			Social	R<0.001, L<0.001, *p*>0.999
		Activity	Individual	R<0.001, L<0.001, *p*>0.999
			Social	R=0.494, L=2.41, *p*=0.121
Crow	1	Frequency	Individual	R<0.001, L<0.001, *p*>0.999
			Social	R<0.001, L<0.001, *p*>0.999
		Activity	Individual	R=0.157, l=0.048, *p*=0.828
			Social	R<0.001, L<0.001, *p*>0.999
	2	Frequency	Individual	R<0.001, L<0.001, *p*>0.999
		Activity	Individual	R<0.001, L<0.001, *p*>0.999
	3	Frequency	Individual	R<0.001, L<0.001, *p*>0.999
		Activity	Individual	R=0.282, l=0.08, *p*=0.772
	4	Frequency	Individual	R=0.102, L=0.113, *p*=0.737
			Social	R<0.001, L<0.001, *p*>0.999
		Activity	Individual	R=0.6, L=2.496, *p*=0.114
			Social	R=0.44, L=0.83, *p*=0.363
	5	Frequency	Individual	R<0.001, L<0.001, *p*>0.999
		Activity	Individual	R=0.264, l=0.245, *p*=0.622
	6	Frequency	Individual	R<0.001, L<0.001, *p*>0.999
		Activity	Individual	R<0.001, L<0.001, *p*>0.999
	7	Frequency	Individual	**R=0.336, L=4.34, *p*=0.037**
			Social	R<0.001, L<0.001, *p*>0.999
		Activity	Individual	R<0.001, L<0.001, *p*>0.999
			Social	**R=0.59, L=4.14, *p*=0.042**
	8	Frequency	Individual	R=0.11, L=0.105, *p*=0.746
			Social	R<0.001, L<0.001, *p*>0.999

		Activity	Individual	R<0.001, L<0.001, p>0.999
			Social	R=0.369, L=1.29, p=0.256
	9	Frequency	Individual	R<0.001, L<0.001, p>0.999
		Activity	Individual	R=0.22, L=0.377, p=0.539
	10	Frequency	Individual	R=0.2, L=0.335, p=0.562
			Social	R<0.001, L<0.001, p>0.999
		Activity	Individual	R=0.15, L=0.179, p=0.672
			Social	R<0.001, L<0.001, p>0.999
