# Supplementary material for: Socially Driven Consistent Behavioural Differences during Development in Common Ravens and Carrion Crows
Source: PLoS One. 2016 Feb 5;11(2):e0148822. doi: 10.1371/journal.pone.0148822 (PMC4746062; doi:10.1371/journal.pone.0148822)
Supplement: S4 Table — (PDF) [file pone.0148822.s004.pdf]

S4 Table. Repeatability over time in individual context in ravens: testing whether, in the ravens, subgroup effects arose from social context or similarity in behaviour to kin. Data from individual context (tested while alone) allocated fictitiously to the same subgroups used in social context tests. Individuals and subgroups did not consistently differ in behaviour; therefore individuals did not behave similarly to their tested subgroup without these conspecifics present. Same rounds included for individual context as previously in social context (rounds 1, 4, 7, 8 and 10; fledging to sub-adult stage). R= repeatability, L = likelihood ratio

**Rounds**	**Measure**	**Individual (WHILE ALONE)/ Subgroup (FICTICIOUS SUBGROUP) effect**	**Novel Food**	**Novel Object**
1-10	Frequency	Individual	R<0.001, L<0.001, p>0.999	R<0.001, L<0.001, p>0.999
1-10	Frequency	Subgroup	R=0.024, L=0.118, p=0.731	R=0.019, L=0.079, p=0.778
1-10	Activity	Individual	R=0.024, L<0.001, p>0.999	R<0.001, L<0.001, p>0.999
1-10	Activity	Subgroup	R<0.001, L<0.001, p>0.999	R<0.001, L<0.001, p>0.999
7-10	Frequency	Individual	R<0.001, L<0.001, p>0.999	R=0.365, l=2.96, p=0.085
7-10	Frequency	Subgroup	R=0.06, L=0.235, p=0.628	R<0.001, L<0.001, p>0.999
7-10	Activity	Individual	R=0.21, L=1.006, p=0.316	R<0.001, L<0.001, p>0.999
7-10	Activity	Subgroup	R=0.07, L=0.007, p=0.932	R<0.001, L<0.001, p>0.999
