# Supplementary material for: Socially Driven Consistent Behavioural Differences during Development in Common Ravens and Carrion Crows
Source: PLoS One. 2016 Feb 5;11(2):e0148822. doi: 10.1371/journal.pone.0148822 (PMC4746062; doi:10.1371/journal.pone.0148822)
Supplement: S5 Table — (PDF) [file pone.0148822.s005.pdf]

S5 Table. Constant homophily models testing for in-group preferences of affiliative, i.e. contact sit interactions in a) sibling groups and b) tested subgroups in ravens and crows. Significant preferences for siblings in both species, though significant preferences for tested subgroup in ravens only. Significant results given in bold.

**a) Sibling groups**	**R-square**	***p***	**Regression coefficients**			
			**Independent**	**Unstandarized coefficient**	**Standarized coefficient**	***p***
**Ravens**	0.335	**0.0041**	Intercept	9.27	0.0	0.996
			In-group	27.93	0.58	**0.0041**
**Crows**	0.191	**0.0087**	Intercept	7.35	0.0	0.9915
			In-group	13.26	0.44	**0.0087**
**b) Tested subgroups**			**Regression coefficients**			
			**Independent**	**Unstandarized coefficient**	**Standarized coefficient**	***p***
**Ravens**	0.299	**0.0027**	Intercept	12.03	0.0	0.998
			In-group	31.47	0.55	**0.0027**
**Crows**	0.023	0.281	Intercept	11.46	0.0	0.86
			In-group	5.54	0.15	0.145
